# Perovskite Solar Cells toward Eco-Friendly Printing

**DOI:** 10.34133/2021/9671892

**Published:** 2021-02-16

**Authors:** Xiaoming Chang, Yuanyuan Fan, Kui Zhao, Junjie Fang, Dongle Liu, Ming-Chun Tang, Dounya Barrit, Detlef-M. Smilgies, Ruipeng Li, Jing Lu, Jianbo Li, Tinghuan Yang, Aram Amassian, Zicheng Ding, Yonghua Chen, Shengzhong (Frank) Liu, Wei Huang

**Affiliations:** ^1^Key Laboratory of Applied Surface and Colloid Chemistry, Ministry of Education, Shaanxi Key Laboratory for Advanced Energy Devices, Shaanxi Engineering Lab for Advanced Energy Technology, School of Materials Science and Engineering, Shaanxi Normal University, Xi'an 710119, China; ^2^King Abdullah University of Science and Technology (KAUST), KAUST Solar Center (KSC) and Physical Science and Engineering Division (PSE), Thuwal 23955-6900, Saudi Arabia; ^3^Cornell High Energy Synchrotron Source, Cornell University, Ithaca, NY 14850, USA; ^4^NSLS II, Brookhaven National Lab, Upton New York 11973, USA; ^5^Department of Materials Science and Engineering, and Carbon and Organic Electronics Laboratories (ORaCEL), North Carolina State University, Raleigh, NC 27695, USA; ^6^Key Laboratory of Flexible Electronics (KLOFE) & Institute of Advanced Materials (IAM), Nanjing Tech University (NanjingTech), Nanjing, 211800 Jiangsu, China; ^7^Dalian National Laboratory for Clean Energy, iChEM, Dalian Institute of Chemical Physics, Chinese Academy of Sciences, Dalian 116023, China; ^8^Frontiers Science Center for Flexible Electronics, Shaanxi Institute of Flexible Electronics (SIFE) and Xi'an Institute of Biomedical Materials & Engineering, Northwestern Polytechnical University (NPU), 127 West Youyi Road, Xi'an, 710072 Shaanxi, China

## Abstract

Eco-friendly printing is important for mass manufacturing of thin-film photovoltaic (PV) devices to preserve human safety and the environment and to reduce energy consumption and capital expense. However, it is challenging for perovskite PVs due to the lack of eco-friendly solvents for ambient fast printing. In this study, we demonstrate for the first time an eco-friendly printing concept for high-performance perovskite solar cells. Both the perovskite and charge transport layers were fabricated from eco-friendly solvents *via* scalable fast blade coating under ambient conditions. The perovskite dynamic crystallization during blade coating investigated using *in situ* grazing incidence wide-angle X-ray scattering (GIWAXS) reveals a long sol-gel window prior to phase transformation and a strong interaction between the precursors and the eco-friendly solvents. The insights enable the achievement of high quality coatings for both the perovskite and charge transport layers by controlling film formation during scalable coating. The excellent optoelectronic properties of these coatings translate to a power conversion efficiency of 18.26% for eco-friendly printed solar cells, which is on par with the conventional devices fabricated *via* spin coating from toxic solvents under inert atmosphere. The eco-friendly printing paradigm presented in this work paves the way for future green and high-throughput fabrication on an industrial scale for perovskite PVs.

## 1. Introduction

Metal-halide hybrid perovskite solar cells (PSCs) have recently emerged as a highly promising photovoltaic candidate on account of their continuously improved efficiency with certified solar cell efficiency surpassing 25% [1]. The performance already rivals those of other thin-film photovoltaic (PV) devices. One significant advantage of PSCs is they can be fabricated *via* simple solution processing using scalable and low-cost vacuum-free PV technologies. It is reported that annual PV installations will rapidly increase from ~650 GW in 2019 to a target of 21.9 TW in 2050 [2]. The corresponding required fast expansion of PV manufacturing demands high-throughput scalable fabrication paradigms for PSCs.

In order to fabricate PSCs on a large scale, scalable deposition of active perovskite and charge transport layers has been developed. Scalable solution deposition methods for perovskite layers have been demonstrated recently, including blade coating, slot die coating, and spray coating [3]. These strategies are compatible with roll-to-roll setups, and a relatively small portion of the precursor ink is wasted in the process. The PCEs of solar cells based on blade-coated perovskite layers have rapidly increased by over 21% [4]. The scalable deposition of charge transport layers is also crucial to scaling up PSCs. Both the inorganic [5–7] and organic charge transport materials [8–10] have been used in PSCs previously, which should have appropriate interfacial energy alignment with the perovskite layer and exhibit compatibility with the solution printing strategy.

One important issue that arises for high-throughput scalable fabrication is the use of toxic solvents. Large amounts of toxic solvents are released during high-throughput continuous deposition and the following postannealing process for the perovskite and charge transport layers. Exposure to toxic solvents in the air of the workplace can be quantified by the workplace exposure limit (WEL). The WEL value is calculated from the time-weighted average (TWA) of the exposure time and can be monitored by the Health and Safety Executive (HSE). From the perspective of human safety in the workplace and environmental issues, exposure to toxic solvents must be regulated. However, toxic solvents have been widely involved in the current reported fabrication processes for perovskite solar cells. For instance, dimethylformamide (DMF) [11–15], gamma-butyrolactone (GBL) [16–18], *N*-methyl-2-pyrrolidone (NMP) [19, 20], and 2-methoxyethanol [21, 22] have been used for deposition of perovskite layers. Other solvents, including toluene or halogenated ones (e.g., chlorobenzene (CB)), are commonly used for the fabrication of the 2,2′,7,7′-tetrakis-(*N*,*N*-di-p-methoxyphenylamine)-9,9′-spirobifluorene (Spiro-OMeTAD) hole transport layer (HTL) or the phenyl-C_61_-butyric acid methyl ester (PC_61_BM) electron transport layer (ETL). These toxic solvents have been selected as the first choice to meet the requirements of favorable morphology of films on relatively small-scale substrates under an inert atmosphere. This morphology is of vital importance for both the perovskite and charge transport layers to realize highly efficient charge generation and extraction. However, exposure to toxic solvents in the air during high-throughput fabrication of PSCs would lead to TWAs far beyond their limits. It is clearly impractical for humans to work in such a workplace. In addition, in order to avoid environmental pollution when using the toxic solvents at an industrial scale, capital expense is required for purification equipment, energy input, and labor for maintenance. Apparently, eco-friendly printing of perovskite solar cells without using toxic solvents is critical to realize industrial-scale fabrication while preserving human health and the environment and reducing energy consumption and capital expense.

Here, we report, for the first time, fast and eco-friendly printing of high-performance perovskite solar cells *via* blade coating the charge transport and perovskite layers under ambient conditions. The perovskite crystallization during blade coating was investigated *in situ* using grazing incidence wide-angle X-ray scattering (GIWAXS). The perovskite crystalline morphology can be regulated *via* changing the processing temperature. The eco-friendly printed solar cells exhibit a power conversion efficiency of 18.26%, which is even above those of their conventional counterparts fabricated *via* spin coating from toxic solvents.

## 2. Results and Discussion

**Figure 1:**
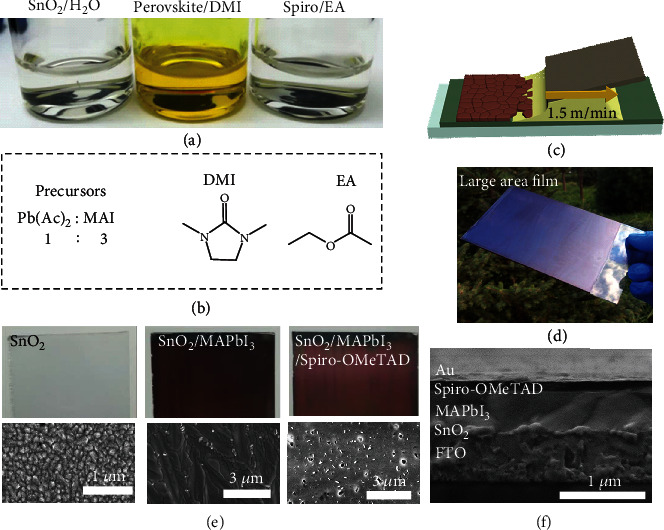
(a) Pictures of SnO_2_ in H_2_O solvent, perovskite precursors in 1,3-dimethyl-2-imidazolidinone (DMI) solvent, and Spiro-OMeTAD in ethyl acetate (EA) solvent. (b) Perovskite precursors and molecular structure of DMI and EA solvents. (c) Schematic illustration of blade coating for perovskite films. (d) Photograph of large-area (20 × 25 cm^2^) MAPbI_3_ films fabricated using the blade coating method. (e) Photographs (top) and scanning electron microscopy (SEM) images (bottom) of SnO_2_, SnO_2_/MAPbI_3_, and SnO_2_/MAPbI_3_/Spiro-OMeTAD films. (f) Cross-sectional SEM image of a complete device.

The solvents H_2_O, 1,3-dimethyl-2-imidazolidinone (DMI), and ethyl acetate (EA) were used to prepare the solutions of SnO_2_ (1.5% mol aqueous solution), perovskite precursors (Pb(Ac)_2_+MAI, 1.2 mol L^−1^), and Spiro-OMeTAD (45 mg mL^−1^), respectively. Photos of the solutions are shown in Figure 1(a), and the corresponding molecular structures of the solvents are illustrated in Figure 1(b). The solvent properties including toxicity, boiling point, WEL, waste issues, fate and effects on the environment, and acute and chronic effects on human health and exposure potential are compared and listed in Table S1 for DMI, EA, and other conventional toxic solvents currently used for perovskite PV manufacturing. Note that we compared tens of solvents and found the DMI solvent to be the best example showing potential for eco-friendly printing of perovskite films because of no substances known to be hazardous to the environment and low volatility (Table S1). Meanwhile, the DMI and EA solvents exhibit much less toxicity to humans or/and much higher WEL than the conventional solvents dimethylformamide (DMF), *N*-methyl-2-pyrrolidone (NMP), 2-methoxyethanol, toluene, and chlorobenzene. The use of lower WEL solvents is beneficial for people working in such a workplace, and it can reduce energy consumption and capital expense for the manufacture of perovskite solar cells under realistic conditions using high-throughput roll-to-roll technology. However, the DMI solvent is a marginal solvent for PbI_2_+MAI precursors and can only be introduced as a solvent additive with a small amount into the DMF system. The resultant PCE of the related solar cells was less than 15% [23]. In contrast, we found that the perovskite precursors Pb(Ac)_2_ and MAI have a high solubility in the DMI solvent, which can be evidenced from the yellow transparent solution (Figure 1(a)). The Spiro-OMeTAD also has a high solubility in the EA solvent. In short, these solvents with low toxicity, high solubility, and high WEL hold promise for the eco-friendly printing of perovskite solar cells. MAPbI_3_ solar cells were realized based on blade-coated perovskite and charge transport layers under ambient conditions with relative humidity (RH) of ca. 35% (Figure 1(c)). Films of SnO_2_, Spiro-OMeTAD, and MAPbI_3_ (except the Au electrode) were fabricated at a high speed of 1.5 m/min, which were used as the ETL, HTL, and photoactive layer, respectively.

A large-scale (20 × 25 cm^2^) sample composed of glass/FTO/SnO_2_/MAPbI_3_ is shown in Figure 1(d). The stacked layers appear uniform and dark without noticeable blemishes, suggesting a high-quality film. This can be further verified from photos of the SnO_2_, SnO_2_/MAPbI_3_, and SnO_2_/MAPbI_3_/Spiro-OMeTAD films (Figure 1(e), top), which are free of pinholes. The scanning electron microscopy (SEM) images show densely packed grains for the SnO_2_ films (Figure 1(e), bottom). In contrast to ~200-300-sized grains for spin-coated MAPbI_3_ films [24], the blade-coated MAPbI_3_ films exhibit grains as large as ~3 *μ*m. Large perovskite grains were also observed previously when blade coating PbI_2_ : MAI precursors from DMSO : GBL solvent or DMF solvent [25]. This morphological difference was attributed to crystallization nucleation and growth during blade coating, which were different from that of conventional spin coating.

The addition of the Spiro-OMeTAD layer fabricated from the EA solvent does not destroy the uniformity of the SnO_2_/MAPbI_3_ films. However, some pinholes with size ~20-50 nm are observed in the Spiro-OMeTAD layer, which was ascribed to the presence of lithium salt, as reported previously [26, 27]. Note that the root-mean-square (RMS) roughness is significantly decreased from 32.10 to 3.77 nm for the SnO_2_/MAPbI_3_ films with the addition of the Spiro-OMeTAD layer (Figure S1). This helps improve contact with the Au electrode and charge extraction layer. Figure 1(f) shows a cross-sectional SEM image of a complete solar cell with the architecture FTO/SnO_2_/perovskite/Spiro-OMeTAD/Au. The thicknesses of the blade-coated SnO_2_, perovskite, and Spiro-OMeTAD layers are approximately 30 nm, 500 nm, and 150 nm, respectively. The perovskite layer is compact without noticeable lateral grain boundaries. These results indicate high-quality films of the three layers blade-coated from eco-friendly solvents.

**Figure 2:**
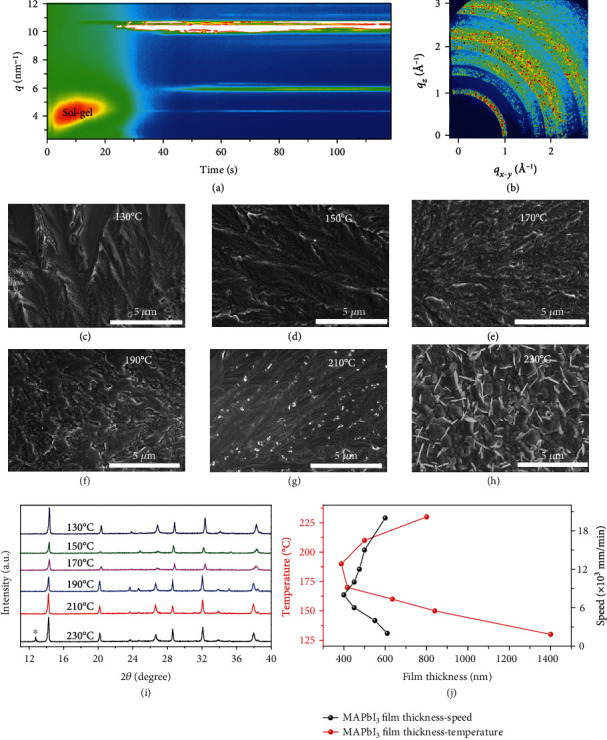
(a) *In situ* GIWAXS analysis showing the dynamic transformation from Pb(Ac)_2_ : MAI precursors in the DMI solvent to perovskite. (b) 2D GIWAXS for the thermally annealed perovskite films. (c–h) SEM images of perovskite films blade-coated at different processing temperatures ranging from 130°C to 230°C. (i) Processing temperature-dependent crystalline features of perovskite films. (j) The influences of processing temperature (fixed speed of 1.5 m/min) and coating speed (fixed temperature of 210°C) on film thickness.

Perovskite crystallization plays a critical role in crystalline morphology and optoelectronic properties. In order to understand how Pb(Ac)_2_ : MAI precursors transition to perovskite during blade coating from the DMI solvent, we first performed *in situ* grazing incidence wide-angle X-ray scattering (GIWAXS) analysis during blade coating. A thin perovskite ink sheet was formed when blade coating was conducted on a room temperature substrate, which has a long solvent evaporation duration longer than 15 min due to the relatively high boiling point of the DMI solvent (225°C). Faster evaporation of the solvent at elevated temperatures allows faster film formation and less moisture attack during perovskite crystallization, which is beneficial for suppressing defects.  Figure 2(a) shows the time evolution of the diffraction features against scattering vector *q* and time (with 0.2 s intervals) when blade coating Pb(Ac)_2_ : MAI precursors on a 150°C substrate. The two-dimensional (2D) snapshots taken at different times are shown in Figure S2. We observed a strong scattering halo at low *q* values of ~2-5 nm^−1^ for the disordered colloidal sol-gel during the initial 30 s. This sol-gel state was observed when solution casting MAI : PbI_2_ precursors from DMF or DMSO : GBL solvents [25, 28–31], during which perovskite precursors in the liquid films grow into the polycrystalline films. Note that the sol-gel window is significantly longer when blade coating precursors from the DMI solvent in contrast to the DMF or DMSO : GBL cases. For instance, the DMF or DMSO : GBL cases exhibit a flash transformation from sol-gel to perovskite within ca. 2-3 s after the blade spreads the solution on a 150°C substrate [25, 30]. In contrast, the DMI case shows a significantly prolonged sol-gel window to ca. 30 s at the same processing temperature. This increase of the sol-gel window is because of less solvent evaporation from the perovskite ink sheet. The sol-gel state is critical for the nucleation and growth of perovskite crystals or intermediates because the fast ionic diffusion determines the assembly behavior between the inorganic framework and the organics including cations and intercalated solvent.

We further observed the formation of intermediate phases at *q* = 4.4 and 5.9 nm^−1^. The formation of intermediate phases indicates a strong interaction between precursors and solvent. However, these intermediate phases are distinct from the previous observation when solution casting MAI : PbI_2_ precursors from DMF or DMSO : GBL solvents. The PbI_2_·DMF and PbI_2_·DMSO solvated intermediate phases exhibit diffraction features at *q* = (4.4, 5.5, and 6.6 nm^−1^) and *q* = (4.4, 5.0, 6.4, and 8.1 nm^−1^) [25, 30], respectively. This indicates that the solvent molecule plays a key role in the crystal structure of the intermediate phases. The diffraction at ~10-11 nm^−1^ accompanied by vanished sol-gel state indicates the formation of MAPbI_3_, which is generally described by the following equation [32]:

(1) $$\begin{array}{c} 3\text{MAI}+\text{Pb}{\left(\text{Ac}\right)}_{2}\to \text{MAPb}{\text{I}}_{3}+2\text{MAAc}. \end{array}$$

Note that intermediate phases are absent after thermal annealing, along with a stronger diffraction intensity of MAPbI_3_ (Figure 2(b)). This indicates that intermediate phases decomplex during thermal annealing and provide a scaffold to initiate further crystallization for released precursors. Meanwhile, the intermediate phases have a higher crystallographic orientation than the MAPbI_3_, which suggests the absence of a template effect provided by intermediate phases on further crystallization.

The influence of the processing temperature on the crystalline morphology of perovskite films was evaluated using scanning electron microscopy (SEM) as shown in Figures 2(c)–2(h) and Figure S3. The films fabricated at a 25°C substrate exhibit isolated islands with a domain size of several micrometers and exposure of the SnO_2_ layer to air (Figure S3). This phenomenon has been also observed previously and well investigated in the case of blade coating MAI : PbI_2_ precursors from DMF or DMSO : GBL solvents on a low-temperature substrate [25, 30], and it was ascribed to the phase transition of sol-gel state → intermediate solvates and/or PbI_2_ crystals → perovskite crystals during film formation. Elevating the processing temperature suppressed the formation of intermediate solvates and/or PbI_2_ crystals, leading to direct transition from the sol-gel state to perovskite crystals. The direct phase transition enabled uniform and compact stacking of perovskite grains. Indeed, we observed improved film uniformity with increasing processing temperature. Note the radial pattern within the polygon grains (Figure S4). Such a phenomenon was attributed to periodic precipitation resulting from solution flow, solute diffusion, solvent drying, and crystal growth [33, 34]. The films blade-coated at 130°C exhibit elongated grains with a domain size of several micrometers with some pinholes interspersed. These pinholes were gradually diminished at higher temperatures. However, PbI_2_ needle crystals were observed when the processing temperature increased to 230°C, suggestive of decomposition of the MAPbI_3_ crystals. This is further verified from X-ray diffraction (XRD) patterns, as shown in Figure 2(i). As a result, we achieved nice films at 210°C, which are free of pinholes and have grain boundaries passivated by some small PbI_2_ crystals with size ~20-50 nm. The self-passivation is beneficial for suppressing defects in the grain boundaries, which in turn decreases the charge recombination loss in a complete device.

The influence of perovskite fluid flow on film formation was further investigated. The meniscus-guided flow during blade coating arises mainly from the capillary flow and viscous forces [35]. The competition between these two driving forces leads to the variation between the evaporation regime for the slow-speed blade coating and the Landau-Levich (LL) regime for the high-speed blade coating [36]. The film thickness obtained at 210°C exhibits an approximate quadratic relation with the coating speed as shown in Figure 2(j). A higher thickness of >800 nm was obtained for both the evaporation and Landau-Levich regimes, while there was a sharp decline to ca. 400 nm at the transition between the two regimes. This decline is because the curvature of the meniscus changes before the contact line in the transition regime, where the liquid is partially dragged out due to the increasingly viscous forces in the presence of solvent evaporation [37]. In the Landau-Levich regime at the coating speed of 1.5 m/min, the contact line is infinitely far away, and therefore, the bulk perovskite liquid sheet is completely dragged out. Note that the processing temperature in the Landau-Levich regime plays a negligible influence on the contact line and the subsequent film thickness (~400-600 nm) because the temperature-dependent solvent evaporation in this regime is negligible. In short, blade coating at a high speed leads to Landau-Levich (LL) flow where the viscous force dominates. In the subsequent quiescent drying stage, the assembly is determined mainly by ionic interactions between precursors because of the absence of meniscus-assisted strain. The sol-gel state, which plays a role in the formation of intermediate solvates and MAPbI_3_ crystals, can be regulated *via* controlling the processing temperature. The films with relatively good uniformity and PbI_2_ passivation are achieved at the processing temperature of 210°C.

**Figure 3:**
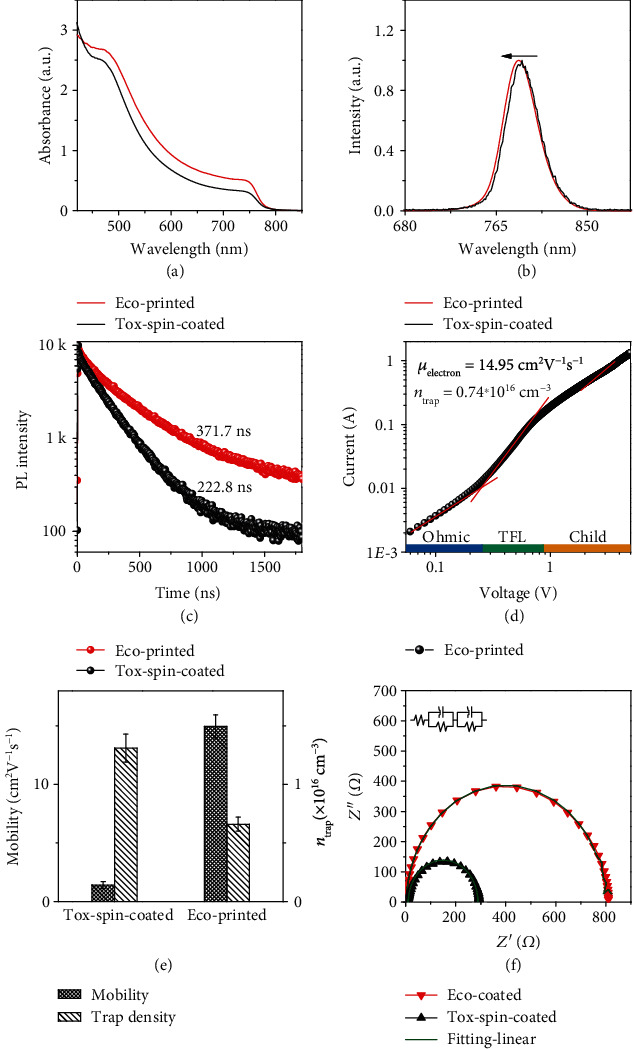
(a) The absorption spectra of eco-printed and tox-spin-coated films. (b) The photoluminescence spectra of eco-printed and tox-spin-coated films. (c) Time-resolved photoluminescence (TRPL) spectra of eco-printed and tox-spin-coated films. (d) Dark *I*-*V* measurement of the electron-only device for the eco-coated films. (e) Statistics of trap densities and electron mobilities of eco-printed and tox-spin-coated films. (f) Electrical impedance spectroscopy (EIS) of eco-printed and tox-spin-coated films.

We carried out a range of complementary characterization measurements to understand the photophysical properties of the perovskite films blade-coated from the DMI solvent (abbreviated as eco-printed). The films fabricated from the toxic solvent DMSO : GBL *via* antisolvent dripping (abbreviated as tox-spin-coated), which has been widely used for lab-scale fabrication, are also shown for comparison. The UV-Vis spectra of the eco-printed and tox-spin-coated films show that, although the former has a higher intensity, they have an identical band edge at ca. 780 nm [38] (Figure 3(a)). This indicates an identical crystal structure irrespective of precursor and solvent properties. This can be further verified from the similar diffraction features located at 14.20° and 28.50° (Figure S5). The eco-printed films exhibit a photoluminescence (PL) peak at ca. 785 nm (Figure 3(b)), which is blue-shifted by 3 nm compared to the tox-spin-coated one. This indicates relatively fewer defects in the eco-printed films partially due to larger grains [39]. Time-resolved PL (TRPL) measurements performed on both the perovskite films help us evaluate the charge carrier dynamics, specific differences in the charge carrier lifetimes (Figure 3(c)). We have used the following biexponential function to determine the lifetimes [40]:

(2) $$\begin{array}{c} f\left(t\right)=A_{1}\exp\left(-\frac{t}{\tau_{1}}\right)+A_{2}\exp\left(-\frac{t}{\tau_{2}}\right)+B, \end{array}$$

where *τ*_1_ and *τ*_2_ are the slow and fast decay time constants, respectively, while *A*_1_ and *A*_2_ are the corresponding decay amplitudes, and *B* is a constant. The carrier dynamics derived from the transient PL behavior provides information about the defect concentration, which is related to nonradiative charge recombination losses. The eco-printed films exhibit an average carrier lifetime (*τ*_ave_) of 371.7 ns, which is even higher than the tox-spin-coated one (ca. 222.8 ns, Table S2). The long carrier lifetime of the eco-printed films indicates a high-quality film, which can be ascribed to large grains and PbI_2_ passivation within the grain boundaries, as reported previously [41].

The trap densities and charge mobilities of the eco-printed films were further evaluated. The dark *I*-*V* characteristics of electron-only devices were obtained based on the architecture glass/FTO/SnO_2_/perovskite/PCBM/Ag and are presented in Figure 3(d) for the blade-coated films. The trap density was determined using the following equation [42]:

(3) $$\begin{array}{c} \;n_{\text{trap}}=\frac{2\varepsilon_{0}\varepsilon_{\text{r}}V_{\text{TFL}}}{{\text{e}L}^{2}}, \end{array}$$

where *ε*_0_ is the vacuum permittivity, *ε*_r_ is the relative dielectric constant, *V*_TFL_ is the onset voltage of the trap-filled limit region, *e* is the elementary charge, and *L* is the distance between the electrodes. The electron mobility was further extracted using the Mott-Gurney law [43]:

(4) $$\begin{array}{c} \mu =\frac{8J_{\text{D}}L^{3}}{9\varepsilon_{0}\varepsilon_{\text{r}}V^{2}}, \end{array}$$

where *J*_D_ is the current density and *V* is the applied voltage. The mobilities were estimated to be 14.95(±1.00) and 1.4(±0.30) cm^2^ V^−1^ s^−1^ for the eco-printed and tox-spin-coated films (Figure 3(e)), respectively. The trap densities are 0.66(±0.06) × 10^16^ and 1.31(±0.12) × 10^16^ cm^−3^ for the eco-printed and tox-spin-coated films, respectively. Apparently, the films fabricated from the eco-friendly solvent under ambient conditions exhibit even higher carrier mobility and lower trap density than those carefully fabricated under inert conditions *via* spin coating from commonly used toxic solvents. These results agree well with the TRPL observation and suggest a low charge recombination loss in a complete solar cell.

The electrical impedance spectroscopy (EIS) of complete devices further provides values of the recombination resistance (*R*_rec_) and the contact resistance (*R*_s_) at the perovskite-contact interface (Figure 3(f)). The Nyquist plots of the tox-spin-coated and eco-printed MAPbI_3_ films were measured under dark conditions at potential biases of 0.8 and 1.0 V, respectively. The *R*_s_ values were determined to be 10.99 and 4.72 *Ω*, along with *R*_rec_ values of 278 and 718 *Ω* for the tox-spin-coated and eco-printed films, respectively (Table S3). The lower *R*_s_ and higher *R*_rec_ values indicate lower charge recombination within the eco-printed films and lower contact resistance at the perovskite-contact interface.

**Figure 4:**
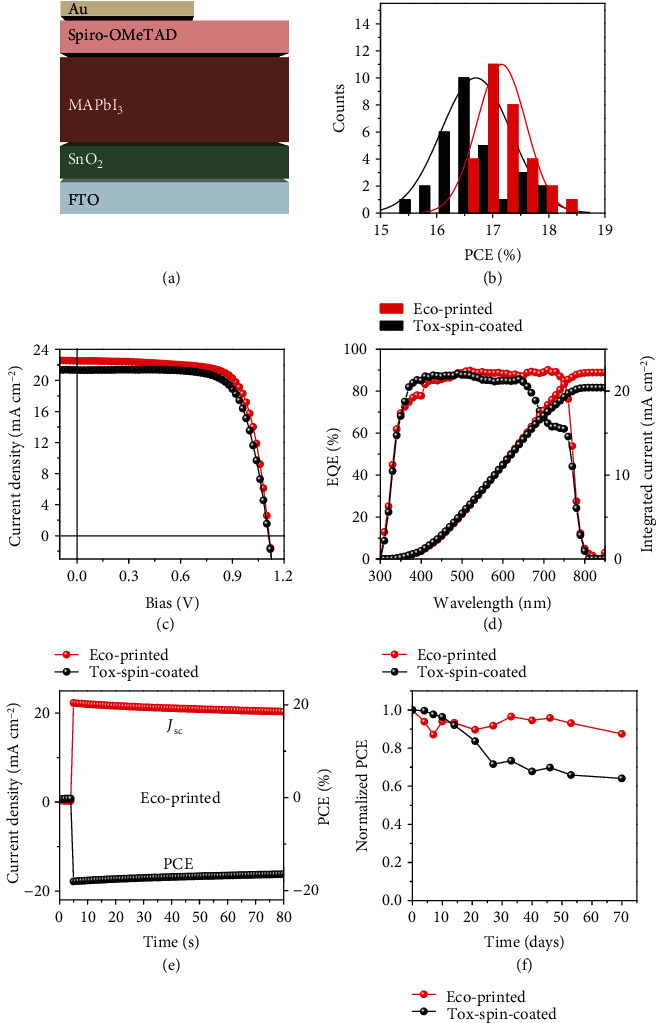
(a) Solar cell architecture. (b) Device performance distributions of 30 eco-printed and tox-spin-coated perovskite solar cells. (c) *J-V* curves of the champion cell for eco-printed and tox-spin-coated solar cells. (d) The external quantum efficiency (EQE) spectrum and the corresponding integrated current for eco-printed and tox-spin-coated perovskite solar cells. (e) The stabilized power output of the eco-printed champion cell measured at a fixed maximum power point (MPP) voltage as a function of time. (f) Comparison of stability of the corresponding nonencapsulated solar cells exposed to the ambient environment with 40-50% humidity in the dark at room temperature.

Solar cells composed of an n-i-p structure were fabricated based on eco-printed SnO_2_, MAPbI_3_, and Spiro-OMeTAD layers (Figure 4(a)). The conventional devices are also shown for comparison and contain a tox-spin-coated MAPbI_3_ layer from DMSO : GBL, a Spiro-OMeTAD layer from chlorobenzene, and a SnO_2_ layer. A histogram of PCEs is shown in Figure 4(b) for two batches of cells with an active size of 0.09 cm^2^. The eco-printed cells achieve PCEs of 17.15 ± 0.44%, which is on par with the tox-spin-coated batches (16.70 ± 0.62%). The champion cell delivers a PCE of 18.26%, a short-circuit current density (*J*_sc_) of 22.52 mA cm^−2^, an open-circuit voltage (*V*_oc_) of 1.11 V, and a fill factor (FF) of 72.89% (Figure 4(c) and Table S4). Typically, the transfer from spin coating in inert conditions to ambient printing results in lower device performance [35]. This behavior is inverted here with the demonstration that the eco-friendly printable solar cells in ambient conditions achieve performance comparable with or higher than the conventional devices tox-spin-coated from toxic solvents in inert conditions (18.26% vs. 17.99%). It is important to note that the high *V*_oc_ of 1.11 V for the eco-friendly printed cells is impressive as this high value is directly related to nonradiative charge recombination losses. These results prove that this eco-friendly printing is suitable for future green and high-throughput fabrication at an industrial scale for high-performance perovskite solar cells.

The external quantum efficiency (EQE) spectra of the corresponding eco-printed and tox-spin-coated cells are evaluated (Figure 4(d)). We observed a higher EQE in the ~650-750 nm range for the eco-printed cell than for the tox-spin-coated cell. This suggests a higher photogenerated current in the eco-printed cell, leading to a higher integrated *J*_sc_ from 20.41 to 22.23 mA cm^−2^ due to the higher carrier mobility for the eco-printed perovskite. The stable output PCE of the champion cell was measured under standard 1 sun illumination (Figure 4(e)) in the air (humidity ~40-50% RH). We observed a drop of the PCE from 18.04% to 16.44% with 80 s of illumination, which highlights an opportunity to further improve the output *via* decreasing the number of surface defects, which are possibly induced by moisture attack during ambient fabrication. It was reported that the interaction between H_2_O and CH_3_NH_3_^+^ is much stronger than that between CH_3_NH_3_^+^ and PbI_3_^−^ [44], which might yield trap formation or halide vacancies after film fabrication and therefore degradation of solar cells. The strategies include surface treatment, additive modification, trap engineering, and dimensional control. Figure 4(f) presents the normalized PCE for the unsealed PSCs after aging for 1680 h in an ambient environment with 40-50% RH at room temperature. The eco-printed device exhibits ambient stability with a 13.5% loss of the initial PCE, which outperforms the conventional tox-spin-coated cell.

In summary, this work demonstrates, for the first time, an eco-friendly printing concept for high-performance perovskite solar cells. High-quality perovskite and charge transport layers were fabricated *via* Landau-Levich flow under ambient conditions from nontoxic and high-WEL solvents. With the aid of *in situ* GIWAXS measurement, we decoupled the phase transition from Pb(Ac)_2_ : MAI precursors to intermediate solvates and perovskite crystals during blade coating from the eco-friendly solvent. The perovskite liquid flow and crystalline morphology can be well regulated *via* controlling the coating speed and processing temperatures, respectively. Compact perovskite films with self-passivation were obtained, which show comparable or even superior optoelectronic properties, including high charge mobility and low trap density compared with the films fabricated *via* spin coating from conventional toxic solvents in inert conditions. The excellent optoelectronic properties finally translate to an unprecedented PCE of 18.26% for solar cells based on eco-friendly printed charge transport and perovskite layers, and this PCE is on par with or even superior to that of the conventional solar cells fabricated *via* spin coating from toxic solvents under inert conditions. Since eco-friendly printing preserves human health and the environment and reduces capital expense, the current work is believed to be helpful for future industrial-scale, green manufacturing of perovskite-based solar cells and electronics without sacrificing device performance.

## 3. Materials and Methods

### 3.1. Material Preparation

CH_3_NH_3_I (MAI, 99.5%) and Pb(Ac)_2_ (99.5%) were purchased from p-OLED. The solvent 1,3-dimethyl-2-imidazolidinone (DMI, 98%) was purchased from Alfa Aesar. Ethyl acetate (EA, 99.9%) and chlorobenzene (CB, 99.9%) were purchased from Acros. The solvents dimethyl sulfoxide (DMSO, 99.8%), *N*,*N*-dimethylformamide (DMF, 99.8%), *γ*-butyrolactone (GBL, 99%), and chlorobenzene (99.8%) were all purchased from Sigma-Aldrich. The Spiro-OMeTAD powder was purchased from Shenzhen Feiming Science and Technology Co., Ltd. Tin (IV) oxide (SnO_2_, 15% in H_2_O colloidal dispersion) was purchased from Alfa.

### 3.2. Solution Preparation

The mixed perovskite precursor was prepared by dissolving a 1.2 mol L^−1^ mixture of metal lead salts, which were composed of 0.39 g Pb(Ac)_2_ and 0.572 g MAI, in a solvent of DMI (1 mL) which was prepared and then stirred at 40°C for 12 hours. The traditional MAPbI_3_ precursor solution was prepared in a glove box in a mixed solvent of DMSO and GBL with a volume ratio of 7 : 3. The solution was filtered prior to solution casting. The Spiro-OMeTAD solution was prepared by dissolving 45 mg of Spiro-OMeTAD, 20 *μ*L of lithium bis(trifluoromethanesulfonyl)imide solution (520 mg in 1 mL acetonitrile), and 30 *μ*L of 4-tert-butylpyridine in 1 mL of ethyl acetate. The SnO_2_ solution was prepared by diluting the original solution ten times. The traditional Spiro-OMeTAD solution was prepared by dissolving 90 mg of Spiro-OMeTAD, 22 *μ*L of lithium bis(trifluoromethanesulfonyl)imide solution (520 mg in 1 mL acetonitrile), and 36 *μ*L of 4-tert-butylpyridine in 1 mL of chlorobenzene.

### 3.3. Device Fabrication

The FTO-coated glass (2.9 cm × 2.9 cm) was cleaned by sequential sonication in acetone, isopropanol, and ethanol for 30 min each and then was dried under N_2_ flow and treated by ozone plasma for 18 min.

### 3.4. The Spin-Coated Films

The TiO_2_ was prepared by chemical bath deposition with the clean substrate immersed in a TiCl_4_ (CP, Sinopharm Chemical Reagent Co., Ltd.) aqueous solution with the volume ratio of TiCl_4_ : H_2_O equal to 0.0225 : 1 at 70°C for 60 min. The spin coating was accomplished under an inert atmosphere inside a nitrogen glove box. The procedure was performed at 1000 rpm for 10 s followed by 4000 rpm for 40 s. At 25 s before the end of the last spin coating step, 250 *μ*L of neat chlorobenzene or loaded solution was dropped onto the substrate, which was then put onto a hot plate for 10 min at 100°C. Subsequently, the Spiro-OMeTAD solution (CB) was deposited on the top of the perovskite by spin coating at 4000 rpm for 15 s followed by evaporation of the 100 nm gold electrode on the top of the cell.

### 3.5. The Eco-Printed Films

The blade coating was conducted in ambient conditions (30-50% RH, 25-30°C). The SnO_2_ solution was drop-cast onto the substrate with a coating speed of 1500 mm min^−1^, and the stage temperature was 100°C. The blade-coated SnO_2_ films were then thermally annealed at 150°C for 30 min. The perovskite precursor solution (6-10 *μ*L) was drop-cast onto the SnO_2_ substrate with a coating speed of 1500 mm min^−1^ at various temperatures. The blade-coated perovskite films were then thermally annealed at 100°C for 7 min. The Spiro-OMeTAD solution (EA) was drop-cast onto the perovskite substrate with a coating speed of 1500 mm min^−1^ at 40°C. The angle between the substrate and the blade was 70°.

### 3.6. Characterizations

#### 3.6.1. Optical Metrology

UV-Vis absorption spectra were acquired on a PerkinElmer UV-Lambda 950 instrument. Steady-state photoluminescence (PL) (excitation at 510 nm, front-side excitation) and time-resolved photoluminescence (TRPL) (excitation at 510 nm, front-side excitation) were measured with a PicoQuant FT300.

#### 3.6.2. Electron Microscopy

The surface morphology and structure of the perovskite films were characterized by SEM (FE-SEM; SU8020, Hitachi).

#### 3.6.3. X-Ray Diffraction (XRD)

The crystal structures of perovskite films were characterized using XRD on Rigaku SmartLab (X-ray source: Cu K*α*, *λ* = 1.54 Å).

#### 3.6.4. Grazing Incidence Wide-Angle X-Ray Scattering Measurements

GIWAXS measurements were performed at D-line of the Cornell High-Energy Synchrotron Source (CHESS). The wavelength of the X-rays was 0.972 Å with a bandwidth Δ*λ*/*λ* of 1.5%. The scattering signal was collected by a Pilatus 200K detector, with a pixel size of 172 *μ*m by 172 *μ*m placed 184.0066 mm away from the sample position. The incidence angle of the X-ray beam was 0.50°. For the blade coating process, the blade passes throughout the substrate spreading the perovskite ink. When the blade is in the frame, it blocks all the scattering signals; the scattering signal reaches the detector as soon as the blade passes and unblocks the path of the beam. The exposure time was kept at 0.2 s to obtain detailed information about the process. Ambient conditions at CHESS were approximately 23°C and ca. 30% relative humidity.

#### 3.6.5. Carrier Mobility Measurements

Electron-only devices (glass/FTO/c-TiO_2_ or SnO_2_/perovskite/PCBM/Ag) were fabricated to measure the electron mobilities of the devices. The dark *J*-*V* characteristics of the electron-only devices were measured using a Keithley 2400 SourceMeter. The mobility was extracted by fitting the *J*-*V* curves in the space-charge-limited current (SCLC) regime with the Mott-Gurney equation. The trap state density was determined from the trap-filled limit voltage using the equation given in the supporting information.

#### 3.6.6. Device Characterization

The *J*-*V* performance of the perovskite solar cells was analyzed using a Keithley 2400 SourceMeter under ambient conditions at room temperature, and the illumination intensity was 100 mW cm^−2^ (AM 1.5G Oriel solar simulator). The scan range was 2 V to -0.1 V. The scan rate was 0.3 V s^−1^. The delay time was 10 ms, and the bias step was 0.02 V. The power output of the lamp was calibrated using an NREL-traceable KG5-filtered silicon reference cell. The device area of 0.09 cm^2^ was defined by a metal aperture to avoid light scattering from the metal electrode into the device during the measurement. The EQE was characterized on a QTest Station 2000ADI system (Crowntech Inc., USA), and the light source was a 300 W xenon lamp. The monochromatic light intensity for the EQE measurement was calibrated with a reference silicon photodiode.

#### 3.6.7. EIS Measurement

EIS measurements were conducted using the electrochemical workstation (IM6ex, Zahner, Germany) with the frequency range from 10 Hz to 4 MHz under 0.8 V bias in the dark.

## Data Availability

The authors declare that in addition to the text and supplementary information, the authors can provide additional supporting data.

## Supplementary Materials

Supplementary Materials Figure S1: AFM images of (a) SnO_2_ films, (b) eco-printed MAPbI_3_ films, and (c) Spiro-OMeTAD films. Figure S2: the two-dimensional (2D) snapshots of eco-printed MAPbI_3_ films taken at different times. Figure S3: (a–f) cross-sectional SEM images of eco-printed MAPbI_3_ films fabricated with substrate temperatures from 130°C to 230°C; (g) top-view SEM images of eco-printed MAPbI_3_ films fabricated on a 25°C substrate; (h) the PCE of MAPbI_3_ PSCs eco-printed on substrates at different temperatures. Figure S4: (a–c) top-view SEM images of eco-printed MAPbI_3_ films fabricated at a 210°C substrate temperature. Figure S5: XRD patterns of tox-spin-coated and eco-printed MAPbI_3_ films. Table S1: the TRPL statistics of the tox-spin-coated and eco-printed perovskite films. Table S2: summaries of EIS parameters for the tox-spin- coated and eco-printed devices. Table S3: the PV performance statistics of the eco-printed PSCs fabricated at different substrate temperatures. Table S4: excerpt of the GSK solvent selection guide for some common solvents for fabricating PSCs.
