# Supplementary material for: Perovskite Solar Cells toward Eco-Friendly Printing
Source: Research (Wash D C). 2021 Feb 16;2021:9671892. doi: 10.34133/2021/9671892 (PMC7906024; doi:10.34133/2021/9671892)
Supplement: Supplementary Materials — Figure S1: AFM images of (a) SnO2 films, (b) eco-printed MAPbI3 films, and (c) Spiro-OMeTAD films. Figure S2: the two-dimensional (2D) snapshots of eco-printed MAPbI3 films taken at different times. Figure S3: (a–f) cross-sectional SEM images of eco-printed MAPbI3 films fabricated with substrate temperatures from 130°C to 230°C; (g) top-view SEM images of eco-printed MAPbI3 films fabricated on a 25°C substrate; (h) the PCE of MAPbI3 PSCs eco-printed on substrates at different temperatures. Figure S4: (a–c) top-view SEM images of eco-printed MAPbI3 films fabricated at a 210°C substrate temperature. Figure S5: XRD patterns of tox-spin-coated and eco-printed MAPbI3 films. Table S1: the TRPL statistics of the tox-spin-coated and eco-printed perovskite films. Table S2: summaries of EIS parameters for the tox-spin- coated and eco-printed devices. Table S3: the PV performance statistics of the eco-printed PSCs fabricated at different substrate temperatures. Table S4: excerpt of the GSK solvent selection guide for some common solvents for fabricating PSCs. [file 9671892.f1.zip › Supporting information.docx]

**Supporting Information**

**Perovskite solar cells towards eco-friendly printing**

Xiaoming Chang,^1^ Yuanyuan Fan,^1^ Kui Zhao,^1^* Junjie Fang,^1^ Dongle Liu,^1^ Ming-Chun Tang,^3^ Dounya Barrit,^3^ Detlef-M. Smilgies,^4^ Ruipeng Li,^5^ Jing Lu,^1^ Jianbo Li,^1^ Tinghuan Yang,^1^ Aram Amassian,^3,6^ Zicheng Ding,^1^ Yonghua Chen,^7^ Shengzhong (Frank) Liu^1,2^*, Wei Huang^8^*

^1^Key Laboratory of Applied Surface and Colloid Chemistry, Ministry of Education; Shaanxi Key Laboratory for Advanced Energy Devices; Shaanxi Engineering Lab for Advanced Energy Technology, School of Materials Science and Engineering, Shaanxi Normal University, Xi’an 710119, China.

^2^Dalian National Laboratory for Clean Energy; iChEM, Dalian Institute of Chemical Physics, Chinese Academy of Sciences, Dalian, 116023, China.

^3^King Abdullah University of Science and Technology (KAUST), KAUST Solar Center (KSC) and Physical Science and Engineering Division (PSE), Thuwal 23955-6900, Saudi Arabia.

^4^Cornell High Energy Synchrotron Source, Cornell University, Ithaca, NY 14850, USA.

^5^NSLS II, Brookhaven National Lab, Upton New York 11973, United States

^6^Department of Materials Science and Engineering, North Carolina State University, Raleigh, NC, 27695, USA.

^7^Key Laboratory of Flexible Electronics (KLOFE) & Institute of Advanced Materials (IAM), Nanjing Tech University (NanjingTech), Nanjing 211800, Jiangsu, China.

^8^Frontiers Science Center for Flexible Electronics, Shaanxi Institute of Flexible Electronics (SIFE) and Xi’an Institute of Biomedical Materials & Engineering, Northwestern Polytechnical University (NPU), 127 West Youyi Road, Xi'an 710072, Shaanxi, China.

**Corresponding Author**

*E-mail: zhaok@snnu.edu.cn, [szliu@dicp.ac.cn and](mailto:szliu@dicp.ac.cn%20and) [provost@nwpu.edu.cn](mailto:provost@nwpu.edu.cn)

**Figure S1.** AFM images of (a) SnO_2_ films (b) Eco-printed MAPbI_3_ films (c) Spiro-OMeTAD films.

**Figure S2.** The two-dimensional (2D) snapshots of eco-printed MAPbI_3_ films taken at different times.

**Figure S3.** (a-f) Cross-sectional SEM images of eco-printed MAPbI_3_ films fabricated with substrate temperatures from 130ºC to 230ºC. (g) Top-view SEM images of eco-printed MAPbI_3_ films fabricated on a 25ºC-substrate. (h) The PCE of MAPbI_3_ PSCs eco-printed on substrates at different temperatures.

**Figure S4.** (a-c) Top-view SEM images of eco-printed MAPbI_3_ films fabricated at a 210ºC substrate temperature.

**Figure S5.** XRD patterns of tox-spin-coated and eco-printed MAPbI_3_ films.

**Table S1.** Excerpt of the GSK solvent selection guide for some common solvents for fabricating PSCs.

| **Solvent** | **Boiling point ℃** | **WEL**  **PPM 8-hour TWA** | **Waste** | **Environ.**  **Impact** | **Health** | **LCA** | **Flag** |
| --- | --- | --- | --- | --- | --- | --- | --- |
| **Ethyl acetate** | **77** | **200** | **4** | **8** | **8** | **6** | **N/L** |
| **1,3-Dimethyl-2-imidazolidinone** | **225** | **N/L** | **5** | **7** | **6** | **N/L** | **N/L** |
| **Chlorobenzene** | **132** | **1** | **6** | **6** | **4** | **8** | **N/L** |
| **DMSO** | **189** | **250** | **5** | **5** | **7** | **6** | **N/L** |
| **DMF** | **153** | **10** | **4** | **6** | **2** | **7** | ***** |
| **NMP** | **203** | **25** | **5** | **6** | **3** | **4** | ***** |
| **2-Methoxyethanol** | **124** | **5** | **3** | **8** | **2** | **7** | **#** |
| **Few Known Issues Some Known Issues Major Known Issues**  **N/L (**Non listed**), WEL (**Workplace exposure limit**), Waste (**Recycling, incineration, volatile organic compound emissions, and biotreatment issues)  **Environ. Impact (**Fate and effects on the environment**), Health (**Acute and chronic effects on human health and exposure potential**)**  **LCA (**Environmental impacts to produce the solvent**), Flag (**Alerts regulatory restrictions**)**  *** (**Must be substituted-a regulatory ban applies**) # (**Substitution recommended-existing regulatory restrictions apply**)** | | | | | | | |

GlaxoSmithKline (GSK) was the first pharmaceutical company to publish a solvent selection guide intended for use in process development. A much abbreviated version of the latest GSK categorization has been provided as **Table S1** listing some common solvents used to fabricate PSCs. The categories are waste, environmental impact, health, life cycle assessment (LCA) and legislative controls (flags). In the table, each of the solvents has a relative ranking from 1 (red) to 10 (green) in seven categories.

In the case of DMI, no formal guidance can be sourced on regulatory WEL values according to Regulation (EC) No. 1907/2006 (REACH), amended by 2015/830/EU. However it will not be classified as hazardous to the aquatic environment according to Regulation (EC) No. 1907/2006 (REACH). Meanwhile, the Exposure Guidelines on the Safety Data Sheet from ThermoFisher Scientific indicate that this product does not contain any hazardous materials with occupational exposure limits established by the region specific regulatory bodies. In conclusion, DMI is a more eco-friendly solvent than chlorobenzene, DMSO, DMF, NMP and 2-Methoxyethanol solvents.

**Table S2.** The TRPL statistics of the tox-spin-coated and eco-printed perovskite films.

| Sample |  | Lifetime τ_1_/ns | Lifetime τ_2_/ns |
| --- | --- | --- | --- |
| Tox-spin-coated | average | 177.4±41.1 | 165.0±39.5 |
|  | max | 222.8 | 207.7 |
| Eco-printed | average | 341.6±29.7 | 283.0±28.5 |
|  | max | 371.7 | 312.4 |

**Table S3.** Summaries of EIS parameters for the tox-spin-printed and eco-printed devices.

| **Sample** | **R_s_ (Ω)** | **C_s_ (F/cm^2^)** | **R_tr_ (Ω)** | **C_rec_ (F/cm^2^)** | **R_rec_ (Ω)** |
| --- | --- | --- | --- | --- | --- |
| Tox-spin-printed | 10.99 | 1.75×10^10^ | 6.93×10^11^ | 1.60×10^-7^ | 278 |
| Eco-printed | 4.72 | 5.39×10^-6^ | 87.69 | 1.99×10^-7^ | 718 |

**Table S4.** The PV performance statistics of the eco-printed PSCs fabricated at different substrate temperatures.

| T (℃) | PCE (%) | *FF* (%) | *J_sc_* (mA cm^-2^) | *V_oc_* (V) |
| --- | --- | --- | --- | --- |
| 25℃ | ×× | ×× | ×× | ×× |
| 150℃ | 6.03±1.78 | 38.77±5.78 | 19.30±0.34 | 0.79±0.15 |
| 170℃ | 13.36±0.87 | 65.27±1.83 | 19.42±0.38 | 0.80±0.04 |
| 190℃ | 15.89±0.56 | 70.82±1.87 | 20.57±0.13 | 0.87±0.02 |
| 210℃ | 17.15±0.44 | 69.98±2.14 | 22.67±0.99 | 1.08±0.02 |
| 230℃ | 9.59±1.47 | 45.79±6.59 | 17.79±2.87 | 0.83±0.35 |
