# Supplementary figures and images for: Perovskite Solar Cells toward Eco-Friendly Printing
Source: Research (Wash D C). 2021 Feb 16;2021:9671892. doi: 10.34133/2021/9671892 (PMC7906024; doi:10.34133/2021/9671892)

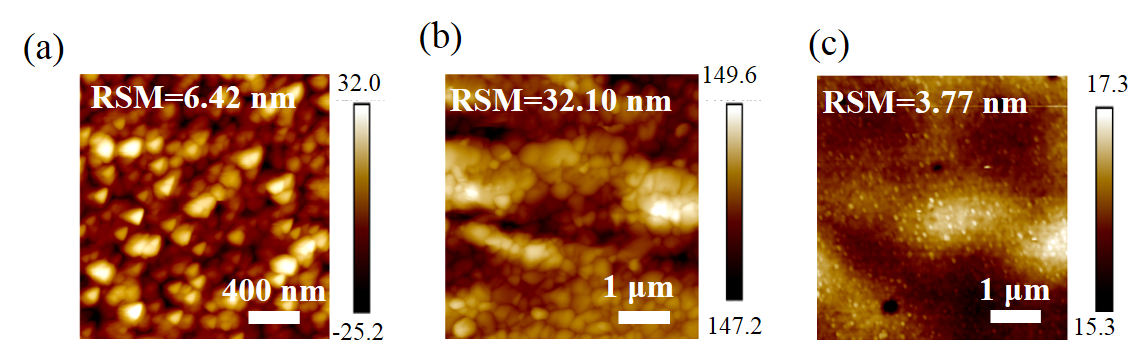

Supplement: Supplementary Materials — Figure S1: AFM images of (a) SnO2 films, (b) eco-printed MAPbI3 films, and (c) Spiro-OMeTAD films. Figure S2: the two-dimensional (2D) snapshots of eco-printed MAPbI3 films taken at different times. Figure S3: (a–f) cross-sectional SEM images of eco-printed MAPbI3 films fabricated with substrate temperatures from 130°C to 230°C; (g) top-view SEM images of eco-printed MAPbI3 films fabricated on a 25°C substrate; (h) the PCE of MAPbI3 PSCs eco-printed on substrates at different temperatures. Figure S4: (a–c) top-view SEM images of eco-printed MAPbI3 films fabricated at a 210°C substrate temperature. Figure S5: XRD patterns of tox-spin-coated and eco-printed MAPbI3 films. Table S1: the TRPL statistics of the tox-spin-coated and eco-printed perovskite films. Table S2: summaries of EIS parameters for the tox-spin- coated and eco-printed devices. Table S3: the PV performance statistics of the eco-printed PSCs fabricated at different substrate temperatures. Table S4: excerpt of the GSK solvent selection guide for some common solvents for fabricating PSCs. [file 9671892.f1.zip › Figure S1.bmp]

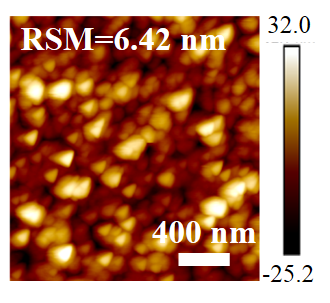

Supplement: Supplementary Materials — Figure S1: AFM images of (a) SnO2 films, (b) eco-printed MAPbI3 films, and (c) Spiro-OMeTAD films. Figure S2: the two-dimensional (2D) snapshots of eco-printed MAPbI3 films taken at different times. Figure S3: (a–f) cross-sectional SEM images of eco-printed MAPbI3 films fabricated with substrate temperatures from 130°C to 230°C; (g) top-view SEM images of eco-printed MAPbI3 films fabricated on a 25°C substrate; (h) the PCE of MAPbI3 PSCs eco-printed on substrates at different temperatures. Figure S4: (a–c) top-view SEM images of eco-printed MAPbI3 films fabricated at a 210°C substrate temperature. Figure S5: XRD patterns of tox-spin-coated and eco-printed MAPbI3 films. Table S1: the TRPL statistics of the tox-spin-coated and eco-printed perovskite films. Table S2: summaries of EIS parameters for the tox-spin- coated and eco-printed devices. Table S3: the PV performance statistics of the eco-printed PSCs fabricated at different substrate temperatures. Table S4: excerpt of the GSK solvent selection guide for some common solvents for fabricating PSCs. [file 9671892.f1.zip › Figure S1a.bmp]

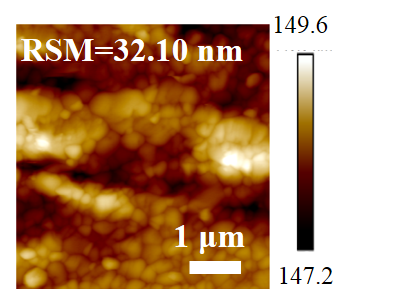

Supplement: Supplementary Materials — Figure S1: AFM images of (a) SnO2 films, (b) eco-printed MAPbI3 films, and (c) Spiro-OMeTAD films. Figure S2: the two-dimensional (2D) snapshots of eco-printed MAPbI3 films taken at different times. Figure S3: (a–f) cross-sectional SEM images of eco-printed MAPbI3 films fabricated with substrate temperatures from 130°C to 230°C; (g) top-view SEM images of eco-printed MAPbI3 films fabricated on a 25°C substrate; (h) the PCE of MAPbI3 PSCs eco-printed on substrates at different temperatures. Figure S4: (a–c) top-view SEM images of eco-printed MAPbI3 films fabricated at a 210°C substrate temperature. Figure S5: XRD patterns of tox-spin-coated and eco-printed MAPbI3 films. Table S1: the TRPL statistics of the tox-spin-coated and eco-printed perovskite films. Table S2: summaries of EIS parameters for the tox-spin- coated and eco-printed devices. Table S3: the PV performance statistics of the eco-printed PSCs fabricated at different substrate temperatures. Table S4: excerpt of the GSK solvent selection guide for some common solvents for fabricating PSCs. [file 9671892.f1.zip › Figure S1b.bmp]

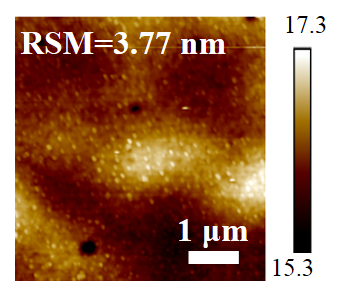

Supplement: Supplementary Materials — Figure S1: AFM images of (a) SnO2 films, (b) eco-printed MAPbI3 films, and (c) Spiro-OMeTAD films. Figure S2: the two-dimensional (2D) snapshots of eco-printed MAPbI3 films taken at different times. Figure S3: (a–f) cross-sectional SEM images of eco-printed MAPbI3 films fabricated with substrate temperatures from 130°C to 230°C; (g) top-view SEM images of eco-printed MAPbI3 films fabricated on a 25°C substrate; (h) the PCE of MAPbI3 PSCs eco-printed on substrates at different temperatures. Figure S4: (a–c) top-view SEM images of eco-printed MAPbI3 films fabricated at a 210°C substrate temperature. Figure S5: XRD patterns of tox-spin-coated and eco-printed MAPbI3 films. Table S1: the TRPL statistics of the tox-spin-coated and eco-printed perovskite films. Table S2: summaries of EIS parameters for the tox-spin- coated and eco-printed devices. Table S3: the PV performance statistics of the eco-printed PSCs fabricated at different substrate temperatures. Table S4: excerpt of the GSK solvent selection guide for some common solvents for fabricating PSCs. [file 9671892.f1.zip › Figure S1c.bmp]

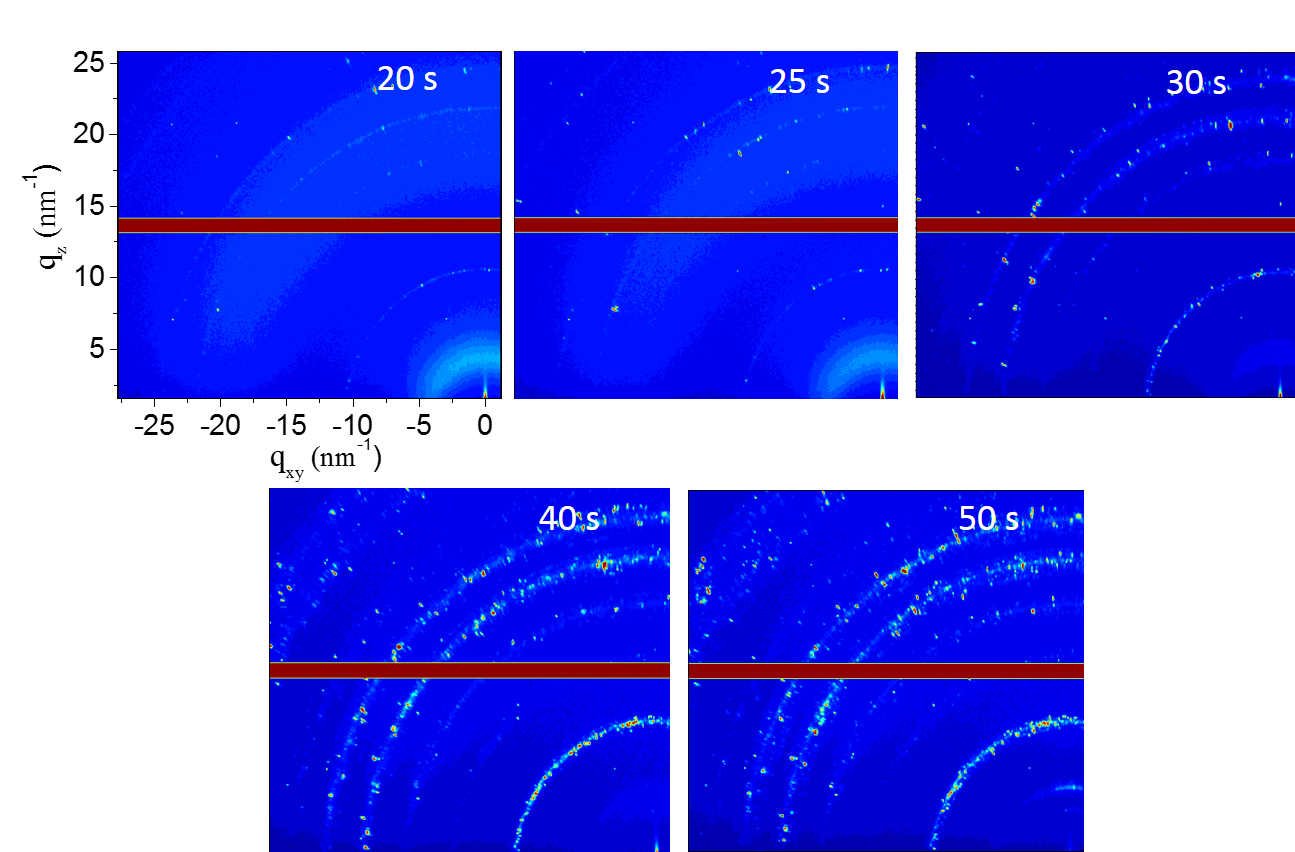

Supplement: Supplementary Materials — Figure S1: AFM images of (a) SnO2 films, (b) eco-printed MAPbI3 films, and (c) Spiro-OMeTAD films. Figure S2: the two-dimensional (2D) snapshots of eco-printed MAPbI3 films taken at different times. Figure S3: (a–f) cross-sectional SEM images of eco-printed MAPbI3 films fabricated with substrate temperatures from 130°C to 230°C; (g) top-view SEM images of eco-printed MAPbI3 films fabricated on a 25°C substrate; (h) the PCE of MAPbI3 PSCs eco-printed on substrates at different temperatures. Figure S4: (a–c) top-view SEM images of eco-printed MAPbI3 films fabricated at a 210°C substrate temperature. Figure S5: XRD patterns of tox-spin-coated and eco-printed MAPbI3 films. Table S1: the TRPL statistics of the tox-spin-coated and eco-printed perovskite films. Table S2: summaries of EIS parameters for the tox-spin- coated and eco-printed devices. Table S3: the PV performance statistics of the eco-printed PSCs fabricated at different substrate temperatures. Table S4: excerpt of the GSK solvent selection guide for some common solvents for fabricating PSCs. [file 9671892.f1.zip › Figure S2.bmp]

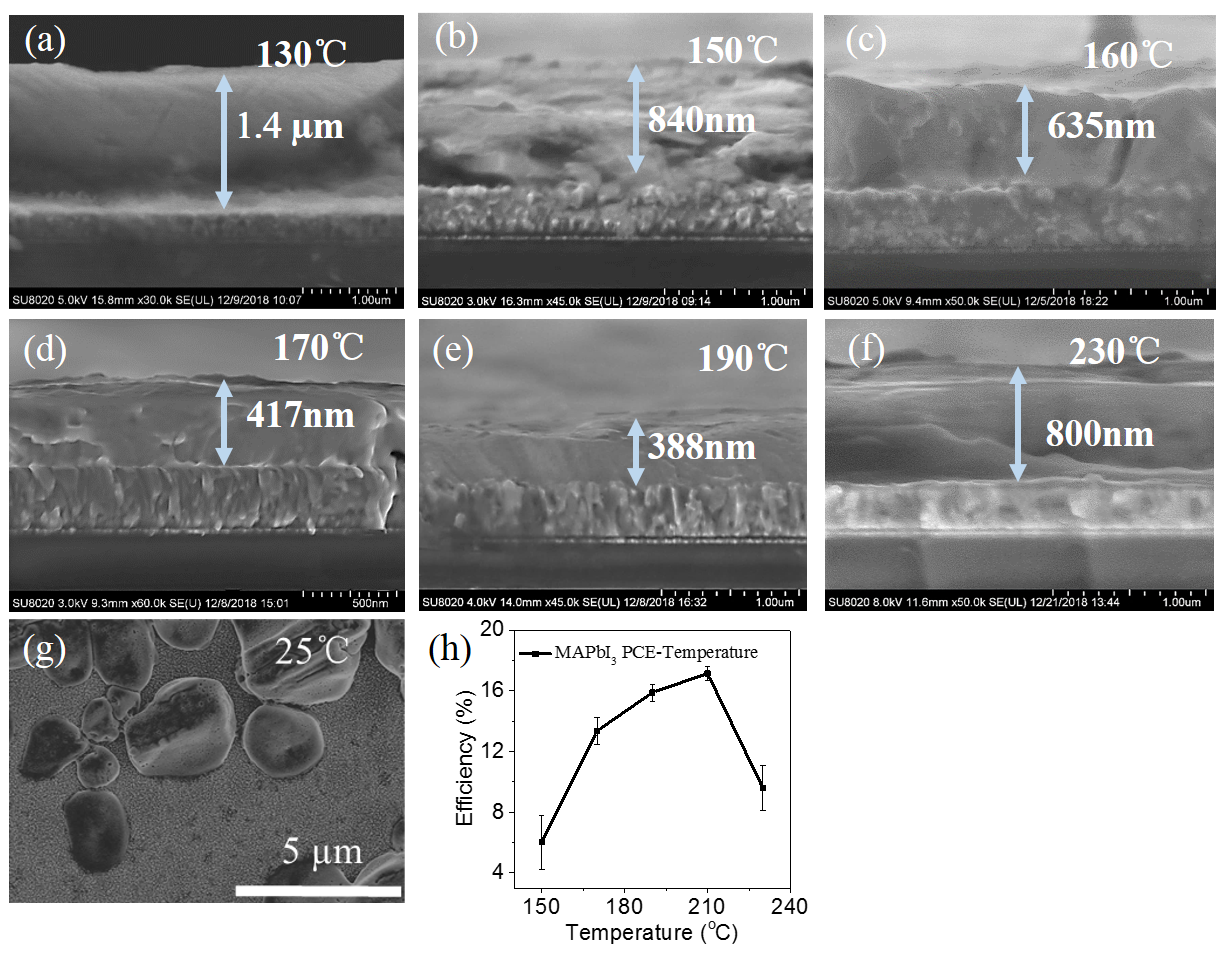

Supplement: Supplementary Materials — Figure S1: AFM images of (a) SnO2 films, (b) eco-printed MAPbI3 films, and (c) Spiro-OMeTAD films. Figure S2: the two-dimensional (2D) snapshots of eco-printed MAPbI3 films taken at different times. Figure S3: (a–f) cross-sectional SEM images of eco-printed MAPbI3 films fabricated with substrate temperatures from 130°C to 230°C; (g) top-view SEM images of eco-printed MAPbI3 films fabricated on a 25°C substrate; (h) the PCE of MAPbI3 PSCs eco-printed on substrates at different temperatures. Figure S4: (a–c) top-view SEM images of eco-printed MAPbI3 films fabricated at a 210°C substrate temperature. Figure S5: XRD patterns of tox-spin-coated and eco-printed MAPbI3 films. Table S1: the TRPL statistics of the tox-spin-coated and eco-printed perovskite films. Table S2: summaries of EIS parameters for the tox-spin- coated and eco-printed devices. Table S3: the PV performance statistics of the eco-printed PSCs fabricated at different substrate temperatures. Table S4: excerpt of the GSK solvent selection guide for some common solvents for fabricating PSCs. [file 9671892.f1.zip › Figure S3.bmp]

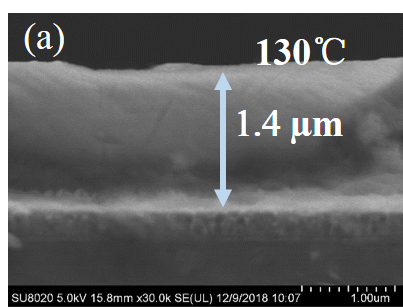

Supplement: Supplementary Materials — Figure S1: AFM images of (a) SnO2 films, (b) eco-printed MAPbI3 films, and (c) Spiro-OMeTAD films. Figure S2: the two-dimensional (2D) snapshots of eco-printed MAPbI3 films taken at different times. Figure S3: (a–f) cross-sectional SEM images of eco-printed MAPbI3 films fabricated with substrate temperatures from 130°C to 230°C; (g) top-view SEM images of eco-printed MAPbI3 films fabricated on a 25°C substrate; (h) the PCE of MAPbI3 PSCs eco-printed on substrates at different temperatures. Figure S4: (a–c) top-view SEM images of eco-printed MAPbI3 films fabricated at a 210°C substrate temperature. Figure S5: XRD patterns of tox-spin-coated and eco-printed MAPbI3 films. Table S1: the TRPL statistics of the tox-spin-coated and eco-printed perovskite films. Table S2: summaries of EIS parameters for the tox-spin- coated and eco-printed devices. Table S3: the PV performance statistics of the eco-printed PSCs fabricated at different substrate temperatures. Table S4: excerpt of the GSK solvent selection guide for some common solvents for fabricating PSCs. [file 9671892.f1.zip › Figure S3a.bmp]

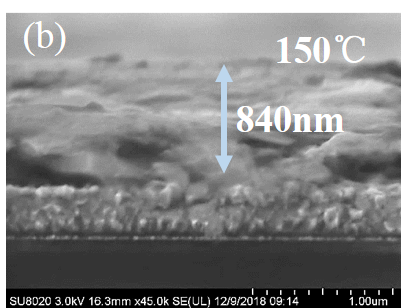

Supplement: Supplementary Materials — Figure S1: AFM images of (a) SnO2 films, (b) eco-printed MAPbI3 films, and (c) Spiro-OMeTAD films. Figure S2: the two-dimensional (2D) snapshots of eco-printed MAPbI3 films taken at different times. Figure S3: (a–f) cross-sectional SEM images of eco-printed MAPbI3 films fabricated with substrate temperatures from 130°C to 230°C; (g) top-view SEM images of eco-printed MAPbI3 films fabricated on a 25°C substrate; (h) the PCE of MAPbI3 PSCs eco-printed on substrates at different temperatures. Figure S4: (a–c) top-view SEM images of eco-printed MAPbI3 films fabricated at a 210°C substrate temperature. Figure S5: XRD patterns of tox-spin-coated and eco-printed MAPbI3 films. Table S1: the TRPL statistics of the tox-spin-coated and eco-printed perovskite films. Table S2: summaries of EIS parameters for the tox-spin- coated and eco-printed devices. Table S3: the PV performance statistics of the eco-printed PSCs fabricated at different substrate temperatures. Table S4: excerpt of the GSK solvent selection guide for some common solvents for fabricating PSCs. [file 9671892.f1.zip › Figure S3b.bmp]

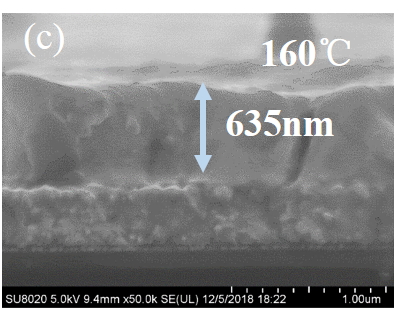

Supplement: Supplementary Materials — Figure S1: AFM images of (a) SnO2 films, (b) eco-printed MAPbI3 films, and (c) Spiro-OMeTAD films. Figure S2: the two-dimensional (2D) snapshots of eco-printed MAPbI3 films taken at different times. Figure S3: (a–f) cross-sectional SEM images of eco-printed MAPbI3 films fabricated with substrate temperatures from 130°C to 230°C; (g) top-view SEM images of eco-printed MAPbI3 films fabricated on a 25°C substrate; (h) the PCE of MAPbI3 PSCs eco-printed on substrates at different temperatures. Figure S4: (a–c) top-view SEM images of eco-printed MAPbI3 films fabricated at a 210°C substrate temperature. Figure S5: XRD patterns of tox-spin-coated and eco-printed MAPbI3 films. Table S1: the TRPL statistics of the tox-spin-coated and eco-printed perovskite films. Table S2: summaries of EIS parameters for the tox-spin- coated and eco-printed devices. Table S3: the PV performance statistics of the eco-printed PSCs fabricated at different substrate temperatures. Table S4: excerpt of the GSK solvent selection guide for some common solvents for fabricating PSCs. [file 9671892.f1.zip › Figure S3c.bmp]

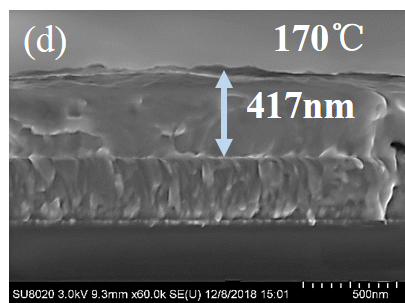

Supplement: Supplementary Materials — Figure S1: AFM images of (a) SnO2 films, (b) eco-printed MAPbI3 films, and (c) Spiro-OMeTAD films. Figure S2: the two-dimensional (2D) snapshots of eco-printed MAPbI3 films taken at different times. Figure S3: (a–f) cross-sectional SEM images of eco-printed MAPbI3 films fabricated with substrate temperatures from 130°C to 230°C; (g) top-view SEM images of eco-printed MAPbI3 films fabricated on a 25°C substrate; (h) the PCE of MAPbI3 PSCs eco-printed on substrates at different temperatures. Figure S4: (a–c) top-view SEM images of eco-printed MAPbI3 films fabricated at a 210°C substrate temperature. Figure S5: XRD patterns of tox-spin-coated and eco-printed MAPbI3 films. Table S1: the TRPL statistics of the tox-spin-coated and eco-printed perovskite films. Table S2: summaries of EIS parameters for the tox-spin- coated and eco-printed devices. Table S3: the PV performance statistics of the eco-printed PSCs fabricated at different substrate temperatures. Table S4: excerpt of the GSK solvent selection guide for some common solvents for fabricating PSCs. [file 9671892.f1.zip › Figure S3d.bmp]

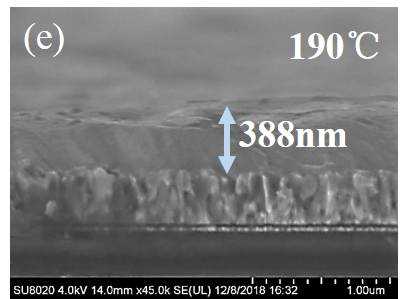

Supplement: Supplementary Materials — Figure S1: AFM images of (a) SnO2 films, (b) eco-printed MAPbI3 films, and (c) Spiro-OMeTAD films. Figure S2: the two-dimensional (2D) snapshots of eco-printed MAPbI3 films taken at different times. Figure S3: (a–f) cross-sectional SEM images of eco-printed MAPbI3 films fabricated with substrate temperatures from 130°C to 230°C; (g) top-view SEM images of eco-printed MAPbI3 films fabricated on a 25°C substrate; (h) the PCE of MAPbI3 PSCs eco-printed on substrates at different temperatures. Figure S4: (a–c) top-view SEM images of eco-printed MAPbI3 films fabricated at a 210°C substrate temperature. Figure S5: XRD patterns of tox-spin-coated and eco-printed MAPbI3 films. Table S1: the TRPL statistics of the tox-spin-coated and eco-printed perovskite films. Table S2: summaries of EIS parameters for the tox-spin- coated and eco-printed devices. Table S3: the PV performance statistics of the eco-printed PSCs fabricated at different substrate temperatures. Table S4: excerpt of the GSK solvent selection guide for some common solvents for fabricating PSCs. [file 9671892.f1.zip › Figure S3e.bmp]

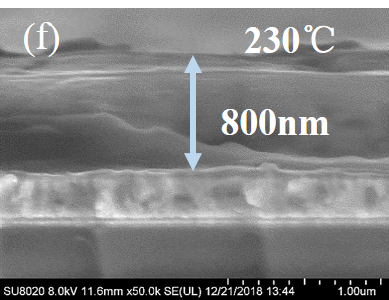

Supplement: Supplementary Materials — Figure S1: AFM images of (a) SnO2 films, (b) eco-printed MAPbI3 films, and (c) Spiro-OMeTAD films. Figure S2: the two-dimensional (2D) snapshots of eco-printed MAPbI3 films taken at different times. Figure S3: (a–f) cross-sectional SEM images of eco-printed MAPbI3 films fabricated with substrate temperatures from 130°C to 230°C; (g) top-view SEM images of eco-printed MAPbI3 films fabricated on a 25°C substrate; (h) the PCE of MAPbI3 PSCs eco-printed on substrates at different temperatures. Figure S4: (a–c) top-view SEM images of eco-printed MAPbI3 films fabricated at a 210°C substrate temperature. Figure S5: XRD patterns of tox-spin-coated and eco-printed MAPbI3 films. Table S1: the TRPL statistics of the tox-spin-coated and eco-printed perovskite films. Table S2: summaries of EIS parameters for the tox-spin- coated and eco-printed devices. Table S3: the PV performance statistics of the eco-printed PSCs fabricated at different substrate temperatures. Table S4: excerpt of the GSK solvent selection guide for some common solvents for fabricating PSCs. [file 9671892.f1.zip › Figure S3f.bmp]

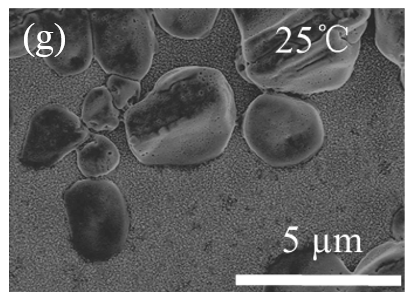

Supplement: Supplementary Materials — Figure S1: AFM images of (a) SnO2 films, (b) eco-printed MAPbI3 films, and (c) Spiro-OMeTAD films. Figure S2: the two-dimensional (2D) snapshots of eco-printed MAPbI3 films taken at different times. Figure S3: (a–f) cross-sectional SEM images of eco-printed MAPbI3 films fabricated with substrate temperatures from 130°C to 230°C; (g) top-view SEM images of eco-printed MAPbI3 films fabricated on a 25°C substrate; (h) the PCE of MAPbI3 PSCs eco-printed on substrates at different temperatures. Figure S4: (a–c) top-view SEM images of eco-printed MAPbI3 films fabricated at a 210°C substrate temperature. Figure S5: XRD patterns of tox-spin-coated and eco-printed MAPbI3 films. Table S1: the TRPL statistics of the tox-spin-coated and eco-printed perovskite films. Table S2: summaries of EIS parameters for the tox-spin- coated and eco-printed devices. Table S3: the PV performance statistics of the eco-printed PSCs fabricated at different substrate temperatures. Table S4: excerpt of the GSK solvent selection guide for some common solvents for fabricating PSCs. [file 9671892.f1.zip › Figure S3g.tif]

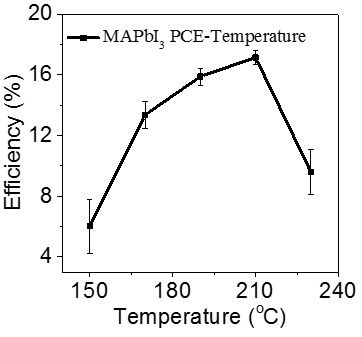

Supplement: Supplementary Materials — Figure S1: AFM images of (a) SnO2 films, (b) eco-printed MAPbI3 films, and (c) Spiro-OMeTAD films. Figure S2: the two-dimensional (2D) snapshots of eco-printed MAPbI3 films taken at different times. Figure S3: (a–f) cross-sectional SEM images of eco-printed MAPbI3 films fabricated with substrate temperatures from 130°C to 230°C; (g) top-view SEM images of eco-printed MAPbI3 films fabricated on a 25°C substrate; (h) the PCE of MAPbI3 PSCs eco-printed on substrates at different temperatures. Figure S4: (a–c) top-view SEM images of eco-printed MAPbI3 films fabricated at a 210°C substrate temperature. Figure S5: XRD patterns of tox-spin-coated and eco-printed MAPbI3 films. Table S1: the TRPL statistics of the tox-spin-coated and eco-printed perovskite films. Table S2: summaries of EIS parameters for the tox-spin- coated and eco-printed devices. Table S3: the PV performance statistics of the eco-printed PSCs fabricated at different substrate temperatures. Table S4: excerpt of the GSK solvent selection guide for some common solvents for fabricating PSCs. [file 9671892.f1.zip › Figure S3h.bmp]

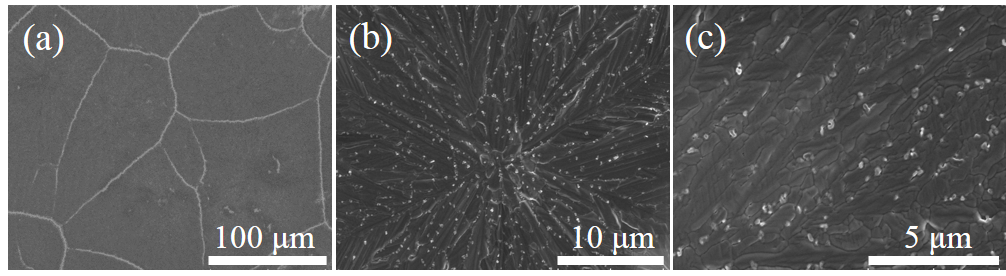

Supplement: Supplementary Materials — Figure S1: AFM images of (a) SnO2 films, (b) eco-printed MAPbI3 films, and (c) Spiro-OMeTAD films. Figure S2: the two-dimensional (2D) snapshots of eco-printed MAPbI3 films taken at different times. Figure S3: (a–f) cross-sectional SEM images of eco-printed MAPbI3 films fabricated with substrate temperatures from 130°C to 230°C; (g) top-view SEM images of eco-printed MAPbI3 films fabricated on a 25°C substrate; (h) the PCE of MAPbI3 PSCs eco-printed on substrates at different temperatures. Figure S4: (a–c) top-view SEM images of eco-printed MAPbI3 films fabricated at a 210°C substrate temperature. Figure S5: XRD patterns of tox-spin-coated and eco-printed MAPbI3 films. Table S1: the TRPL statistics of the tox-spin-coated and eco-printed perovskite films. Table S2: summaries of EIS parameters for the tox-spin- coated and eco-printed devices. Table S3: the PV performance statistics of the eco-printed PSCs fabricated at different substrate temperatures. Table S4: excerpt of the GSK solvent selection guide for some common solvents for fabricating PSCs. [file 9671892.f1.zip › Figure S4.bmp]

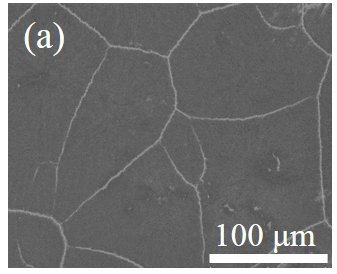

Supplement: Supplementary Materials — Figure S1: AFM images of (a) SnO2 films, (b) eco-printed MAPbI3 films, and (c) Spiro-OMeTAD films. Figure S2: the two-dimensional (2D) snapshots of eco-printed MAPbI3 films taken at different times. Figure S3: (a–f) cross-sectional SEM images of eco-printed MAPbI3 films fabricated with substrate temperatures from 130°C to 230°C; (g) top-view SEM images of eco-printed MAPbI3 films fabricated on a 25°C substrate; (h) the PCE of MAPbI3 PSCs eco-printed on substrates at different temperatures. Figure S4: (a–c) top-view SEM images of eco-printed MAPbI3 films fabricated at a 210°C substrate temperature. Figure S5: XRD patterns of tox-spin-coated and eco-printed MAPbI3 films. Table S1: the TRPL statistics of the tox-spin-coated and eco-printed perovskite films. Table S2: summaries of EIS parameters for the tox-spin- coated and eco-printed devices. Table S3: the PV performance statistics of the eco-printed PSCs fabricated at different substrate temperatures. Table S4: excerpt of the GSK solvent selection guide for some common solvents for fabricating PSCs. [file 9671892.f1.zip › Figure S4a.bmp]

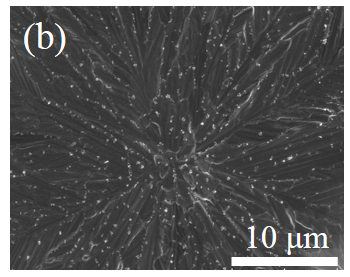

Supplement: Supplementary Materials — Figure S1: AFM images of (a) SnO2 films, (b) eco-printed MAPbI3 films, and (c) Spiro-OMeTAD films. Figure S2: the two-dimensional (2D) snapshots of eco-printed MAPbI3 films taken at different times. Figure S3: (a–f) cross-sectional SEM images of eco-printed MAPbI3 films fabricated with substrate temperatures from 130°C to 230°C; (g) top-view SEM images of eco-printed MAPbI3 films fabricated on a 25°C substrate; (h) the PCE of MAPbI3 PSCs eco-printed on substrates at different temperatures. Figure S4: (a–c) top-view SEM images of eco-printed MAPbI3 films fabricated at a 210°C substrate temperature. Figure S5: XRD patterns of tox-spin-coated and eco-printed MAPbI3 films. Table S1: the TRPL statistics of the tox-spin-coated and eco-printed perovskite films. Table S2: summaries of EIS parameters for the tox-spin- coated and eco-printed devices. Table S3: the PV performance statistics of the eco-printed PSCs fabricated at different substrate temperatures. Table S4: excerpt of the GSK solvent selection guide for some common solvents for fabricating PSCs. [file 9671892.f1.zip › Figure S4b.bmp]

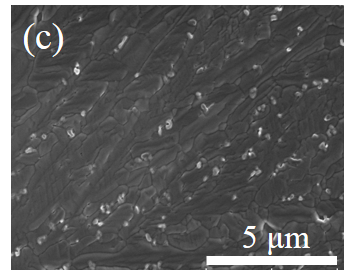

Supplement: Supplementary Materials — Figure S1: AFM images of (a) SnO2 films, (b) eco-printed MAPbI3 films, and (c) Spiro-OMeTAD films. Figure S2: the two-dimensional (2D) snapshots of eco-printed MAPbI3 films taken at different times. Figure S3: (a–f) cross-sectional SEM images of eco-printed MAPbI3 films fabricated with substrate temperatures from 130°C to 230°C; (g) top-view SEM images of eco-printed MAPbI3 films fabricated on a 25°C substrate; (h) the PCE of MAPbI3 PSCs eco-printed on substrates at different temperatures. Figure S4: (a–c) top-view SEM images of eco-printed MAPbI3 films fabricated at a 210°C substrate temperature. Figure S5: XRD patterns of tox-spin-coated and eco-printed MAPbI3 films. Table S1: the TRPL statistics of the tox-spin-coated and eco-printed perovskite films. Table S2: summaries of EIS parameters for the tox-spin- coated and eco-printed devices. Table S3: the PV performance statistics of the eco-printed PSCs fabricated at different substrate temperatures. Table S4: excerpt of the GSK solvent selection guide for some common solvents for fabricating PSCs. [file 9671892.f1.zip › Figure S4c.bmp]

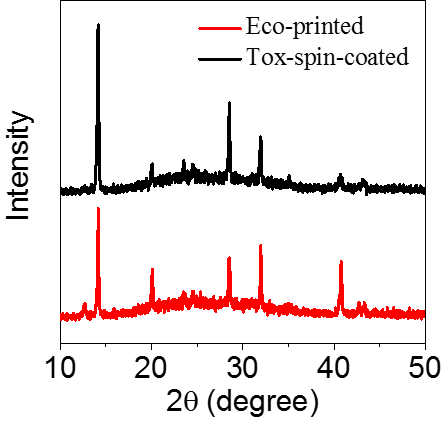

Supplement: Supplementary Materials — Figure S1: AFM images of (a) SnO2 films, (b) eco-printed MAPbI3 films, and (c) Spiro-OMeTAD films. Figure S2: the two-dimensional (2D) snapshots of eco-printed MAPbI3 films taken at different times. Figure S3: (a–f) cross-sectional SEM images of eco-printed MAPbI3 films fabricated with substrate temperatures from 130°C to 230°C; (g) top-view SEM images of eco-printed MAPbI3 films fabricated on a 25°C substrate; (h) the PCE of MAPbI3 PSCs eco-printed on substrates at different temperatures. Figure S4: (a–c) top-view SEM images of eco-printed MAPbI3 films fabricated at a 210°C substrate temperature. Figure S5: XRD patterns of tox-spin-coated and eco-printed MAPbI3 films. Table S1: the TRPL statistics of the tox-spin-coated and eco-printed perovskite films. Table S2: summaries of EIS parameters for the tox-spin- coated and eco-printed devices. Table S3: the PV performance statistics of the eco-printed PSCs fabricated at different substrate temperatures. Table S4: excerpt of the GSK solvent selection guide for some common solvents for fabricating PSCs. [file 9671892.f1.zip › Figure S5.bmp]
